# Multidisciplinary Approach to the Diagnosis of Occult Primary Neuroendocrine Neoplasm: A Clinical Challenge

**DOI:** 10.3390/jcm12175537

**Published:** 2023-08-25

**Authors:** Roberta Elisa Rossi, Francesca Corti, Sara Pusceddu, Massimo Milione, Jorgelina Coppa, Benedetta Masoni, Simone Oldani, Giovanna Sabella, Pietro Cafaro, Alessandro Repici

**Affiliations:** 1 Gastroenterology and Endoscopy Unit, IRCCS Humanitas Research Hospital, Via Manzoni 56, Rozzano, 20089 Milan, Italy; benedetta.masoni@humanitas.it (B.M.); alessandro.repici@hunimed.eu (A.R.); 2 Medical Oncology Unit, Fondazione IRCCS San Gerardo dei Tintori Monza, Via G.B. Pergolesi, 20900 Monza, Italy; francesca.corti@irccs-sangerardo.it (F.C.); p.cafaro@unibs.it (P.C.); 3 Gastro-Entero-Pancreatic and Neuroendocrine Tumor Unit 1, Department of Medical Oncology, ENETS Center of Excellence, Fondazione IRCCS Istituto Nazionale dei Tumori, Via Venezian 1, 20133 Milan, Italy; sara.pusceddu@istitutotumori.mi.it (S.P.); simone.oldani@istitutotumori.mi.it (S.O.); 4 Department of Pathology and Laboratory Medicine, Fondazione IRCCS–Istituto Nazionale dei Tumori, 20133 Milan, Italy; massimo.milione@istitutotumori.mi.it (M.M.); giovanna.sabella@istitutotumori.mi.it (G.S.); 5 Hepatology and Hepato-Pancreatic-Biliary Surgery and Liver Transplantation, Fondazione IRCCS Istituto Nazionale Tumori, Via Venezian 1, 20133 Milan, Italy; jorgelina.coppa@istitutotumori.mi.it; 6 Department of Biomedical Sciences, Humanitas University, Via Rita Levi Montalcini 4, Pieve Emanuele, 20090 Milan, Italy

**Keywords:** neuroendocrine neoplasms, unknown primary tumor, diagnosis, ultrasound endoscopy, capsule endoscopy, double-balloon enteroscopy, treatment, immunohistochemistry, molecular biology

## Abstract

Approximately 11% to 14% of subjects with neuroendocrine neoplasms (NENs) have metastatic lesions with unknown primary origin (UPO), with the majority of UPO-NENs found in the small bowel. Herein, we assessed the available literature on UPO-NENs, focusing on clinical presentation and diagnostic techniques to identify the primary site. The identification of the primary tumor is important as it affects the prognosis; however, the clinical presentation can be non-specific in non-functioning forms. In the presence of metastatic disease, the histological sample is fundamental to obtain immunohistochemical markers that might orientate the clinician in the search for the primary tumor through radiology, functional imaging and endoscopic techniques. In summary, multidisciplinary management plays a key role in UPO-NENs, even more than in other NENs. Molecular biology and gene-expression profiling represent areas of great interest which might be developed in the near future for both the diagnosis and the treatment of these neoplasms.

## 1. Introduction

Neuroendocrine neoplasms (NENs) are rare tumors that originate in diffuse neuroendocrine cells, potentially affecting any organ. NENs encompass a large and heterogenous group of neoplasms characterized by different biological behavior, depending on the clinical and histopathological features and primary site. NENs are classified into well-differentiated G1–G3 NENs and poorly differentiated G3 neuroendocrine carcinomas (NECs), based on their morphological features and proliferation rate. This dichotomous morphological classification reflects underlying differences in terms of genomic characteristics, clinical and biological behavior. We define NENs of unknown primary origin (UPO-NENs) whenever there is a histologically confirmed metastatic disease without an identifiable primary tumor. The metastatic sites associated with UPO-NENs are the liver, followed by the peritoneum, the lymph nodes and, less often, the bones and the lung [1].

While many studies explore differential NEN outcomes according to their origin, only a few studies have evaluated the outcomes of UPO-NENs. An unknown primary site can be considered a poor prognostic factor, especially for patients with advanced-stage disease [2]. However, these cancers can be stratified into favorable (approximately 20%) and poor (approximately 80%) prognostic groups, based on clinical presentation, host factors, tumor histology, functionality, disease burden, location of metastatic sites and sensitivity to chemoradiation treatment. Patients with UPO-NENs have an overall survival of 6 to 9 months, although the favorable prognostic group may have a median survival of nearly 36 months [3]. Early localization of the primary tumor is of utmost importance to define the patient’s management and prognosis [4]. As a matter of fact, prompt identification of the primary tumor site improves the clinical outcome as, according to available evidence [5,6], the resection of the primary tumor also improves survival in in the presence of liver metastases. An Italian retrospective study [6] including 139 liver-metastatic well-differentiated NENs reported that primary tumor resection was an independent positive prognostic factor in multivariate analysis; notably, also in the group of 103 patients with non-resectable liver metastases, the resection of the primary tumor was significantly associated with prolonged survival. In fact, limiting the disease to the liver allows several potentially curative treatment options, including liver resection or liver transplant (in cases of tumors originating from the gastro-entero-pancreatic tract). Furthermore, the resection of the primary tumor reduces the risk of local complication, particularly in the case of small-bowel NENs, such as occlusion, perforation and/or bleeding [6]. Finally, surgery of the primary tumor allows for a biological assessment of the disease and access to potential treatments which require the primary tumor to be clearly identified.

Based on the above observations, in the current review we assessed the available literature on UPO-NENs, focusing on clinical presentation and diagnostic techniques to identify the primary site (radiological/metabolic imaging, endoscopic procedures and molecular pathology), highlighting the need for prompt identification of the primary tumor site and also providing a potential diagnostic algorithm.

## 2. Materials and Methods

A bibliographical search was performed in PubMed to identify guidelines and the primary literature (retrospective and prospective studies, systematic reviews, case series) published in the last 15 years, using both medical subject heading (MeSH) terms and free-language keywords: neuroendocrine neoplasms; unknown primary tumor; diagnosis; ultrasound endoscopy; capsule endoscopy; double-balloon enteroscopy; treatment; immunohistochemistry; molecular biology. The reference lists from the studies returned by the electronic search were manually searched to identify further relevant reports. Articles published as abstracts were included, whereas non-English-language papers were excluded.

## 3. Results

**Figure 1:**
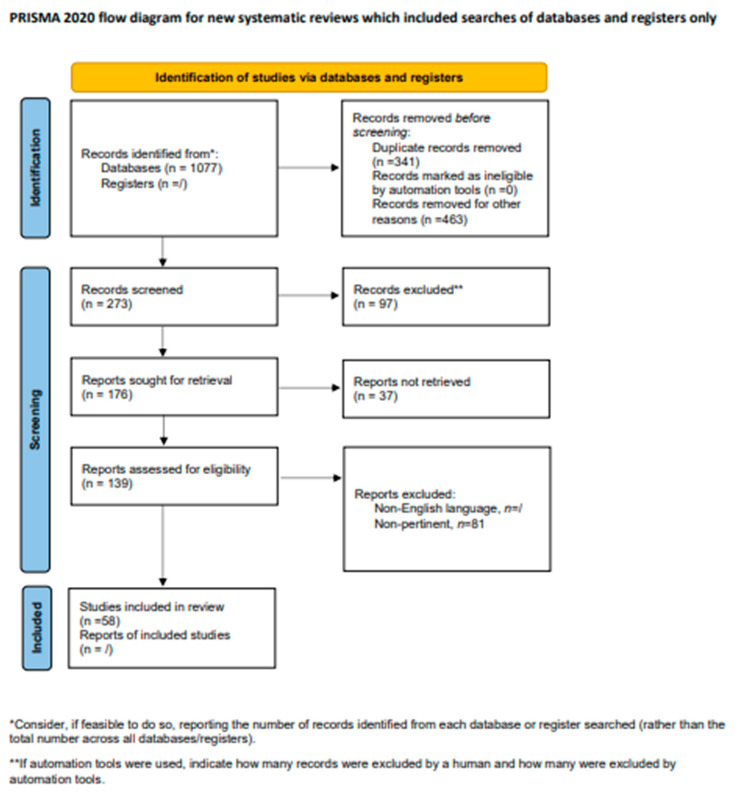
Flow chart showing the process of study selection.

A total of 139 records were reviewed and 58 were defined as fulfilling the criteria for final consideration. Figure 1 presents a flow chart showing the process of study selection.

### 3.1. Epidemiology

NENs represent around 0.5% of all newly diagnosed neoplasms [7]. In recent decades, the incidence of NENs has hugely increased, likely due to improvements in diagnostic techniques and increased disease awareness [2], being approximately 5.86/100,000 per year [8]. The most frequent primary sites are represented by the gastrointestinal/pancreatic tract (62–67%) and lung (22–27%). In well-differentiated tumors, the majority of metastatic sites are found within the liver only [7].

Approximately 11% to 14% of subjects with NENs present metastatic lesions with a UPO, being the majority of UPO-NENs found in the small bowel [9], particularly for well-differentiated forms, followed by the pancreas. Conversely, in poorly differentiated forms, the primary site is generally located in the lung [10]. In 2020, Abdel-Rahman et al. [11] conducted a real-world, population-based study to evaluate the actual incidence and outcome of UPO-NENs. Out of a total of 51,415 recorded cases with NENs, a total of 3550 cases (7%) were diagnosed with UPO-NENs. The authors observed first that the diagnosis of UPO-NENs has increased across the past 4 decades; furthermore, they reported that metastatic small-intestinal NENs appear to have a better prognosis when compared with metastatic UPO-NENs (for both carcinoid tumors and neuroendocrine carcinomas).

### 3.2. Clinical Presentation

In the neuroendocrine setting, the majority of symptoms are non-specific and tend to overlap with more common, often gastro-intestinal (GI), conditions, leading to a significant delay in diagnosis. This assumption is particularly true for those cases in which the primary lesion is undetectable thorough conventional imaging techniques [computed tomography (CT) scan, magnetic resonance imaging (MRI)], and the diagnosis of NENs may be, therefore, mistakenly shelved in favor of other endocrine or GI disorders contributing to the aforementioned diagnostic delay.

Clinical features may be related to the tumor’s hormonal production (functioning NENs), to the site of the primary tumor or to its metastases (mostly hepatic). Functioning NENs can be responsible for many renowned clinical syndromes (as depicted in Table 1), while non-functioning forms’ presentation is often connected to their mass effect.

Midgut/small-intestine NENs (SI-NENs), generally represent the majority of UPO-NENs, which account for 12–22% of all patients diagnosed with NENs [12].

In this scenario, frequent local symptoms include: bowel obstruction or perforation (as a matter of fact, small-bowel NENs are often identified during emergency abdominal surgery), obscure intestinal bleeding without any significative endoscopic finding, unexplained anemia from chronic blood loss or, rarely, obstructive manifestations from vascular compression. Likewise, occult bronchial NENs can be responsible for hemoptysis, dyspnea or recurrent infections due to bronchial obstruction.

The presence of liver metastases can be symptomatic itself by causing abdominal pain (due to liver-capsule stretching or bleeding) or mixed hyperbilirubinemia (as a result of both obstruction and hepatic failure) up to obstructive jaundice. In addition, liver metastases—whether detectable through conventional imaging or not [13]—can be responsible for the development of carcinoid syndrome (CS), a clinical syndrome characterized by flushing, diarrhea and bronchospasm as leading symptoms that can lead to life-threatening complications, such as carcinoid heart disease. The prevalence of CS in patients with NENs has grown significantly in the past decade together with the well-known increase in NENs’ incidence: a large American study showed an increase in its incidence from 11% to 19% during the decade 2000–2011 and its association mainly to midgut NENs (40%); moreover, the presence of CS seemed to be linked to a shorter overall survival [14]. In the setting of UPO-NENs, CS can represent the first or the only clinical manifestation (especially if the primary tumor has a small size), but, again, its symptoms can be mistaken for other conditions (including anxiety, irritable bowel syndrome, menopause, allergic asthma) and the presence of liver metastases frequently ends up being an incidental finding. It is, indeed, a common experience that the diagnosis of NENs is generally delayed and patients with small-bowel NENs are often erroneously diagnosed with irritable bowel syndrome or inflammatory bowel disease due to the non-specific clinical presentation.

### 3.3. Diagnostic Work-Up

Localization of midgut tumors might be challenging due to their usually small size. Early localization of the primary site is a fundamental prerequisite for improving the patient’s management and prolonging survival [4], especially for patients with well-differentiated NENs.

A continuum of investigations to identify the primary tumor is warranted. A multimodal imaging approach, including CT, MRI, positron emission tomography (PET) and somatostatin receptor scintigraphy (SRS) together with endoscopy, is often necessary for detecting the primary tumor [15,16]. In addition to conventional upper and lower GI endoscopy, more sophisticated techniques, including CT enterography, CT angiography, video capsule endoscopy or double-balloon enteroscopy and endoscopic ultrasonography, may all be combined to shed light on challenging cases [16,17]. In selected cases, whenever all the available diagnostic tools have failed, surgical exploration may be warranted. In this setting, an open exploration is considered to be superior to laparoscopy when the primary site cannot be identified but the data are limited [17,18]. However, despite surgical exploration, the primary site is not found in approximately 13% of the cases [16].

The presence of a functional syndrome might be of help to identify the site of the primary lesion in UPO-NENs. In fact, CS is typically secondary to an NEN located in the small bowel and, in this setting, 5-hydroxyindoleacetic acid (5-HIAA) urine levels should be determined, being the specific biomarker for CS [19]. On the other hand, when a functioning NEN as a gastrinoma is suspected, the primary lesion is generally small in size, difficult to be detected and often located at an anatomic region known as the gastrinoma triangle [20]. In the presence of paraneoplastic syndrome, including ectopic ACTH syndrome, a primary tumor located in the lung, the thyroid (medullary carcinoma) or associated with a gastrinoma should be suspected [21]. However, specific biomarkers for UPO-NENs are still lacking.

In clinical practice, the first sign of a neoplastic process secondary to a UPO-NEN is the detection of liver metastases via conventional radiology (i.e., CT scan). Additional work-up, such as upper and lower GI endoscopy, chest CT and MRI of the abdomen, should be required. Conventional radiology might fail to detect the primary tumor in the pancreas or small bowel when the lesions are small or the tests are performed using a suboptimal protocol [22].

#### 3.3.1. Pathology

In patients with UPOs, immunohistochemical markers are useful for cell-type determination and pathologic diagnosis.

**Figure 2:**
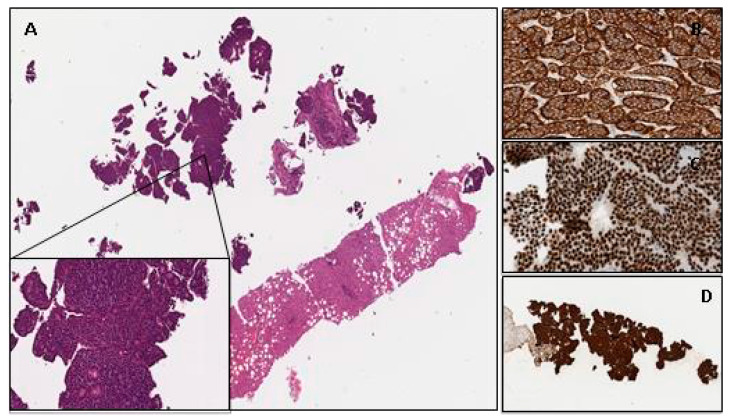
Histological and immunohistochemical attributes of a metastatic small-intestinal NET (SI-NET). (**A**), Characteristic organoid or nested architectural pattern of well-differentiated neuroendocrine tumor. Immunohistochemical expression was noted for Chromogranin (**A**,**B**), CDX2 (**C**) and Serotonin (**D**).

UPO-NENs are most often well-differentiated grade 1 or 2 tumors which commonly originate from the intestinal or pancreatic system (approximately 60–65% of cases) or lungs (approximately 20–25%) [7,23]. Liver metastases dominate in the clinical setting, and these lesions are usually reachable using a core-needle biopsy (CNB), as current guidelines strongly recommend; however, occasionally, focal liver resections might be necessary to obtain sufficient material [24]. An example from our experience is as follows: a 50-year-old man’s biopsy with a single liver metastasis in apparently occult primary tumor is shown in Figure 2. The histopathological findings of the specimen revealed a well-differentiated neoplasm with a predominantly nested architecture on routine hematoxylin–eosin stain (A), monotonous small-sized cells with round nuclei, finely stippled chromatin and heavy eosinophilic cytoplasmic granularity diagnostic of an enterochromaffin-cell (EC) tumor; 100% of the neoplastic cells showed a strong cytoplasmatic immunoreactivity for general neuroendocrine markers such as Chromogranin-A (B) and Synaptophisin as well as for small intestine site-specific immunohistochemical markers such as *CDX2* (C) and serotonin (D); tumors with this morphology and immunohistochemical profile typically arise in the ileum (metastatic small-intestine well-differentiated NEN, SI-WDNEN).

**Figure 3:**
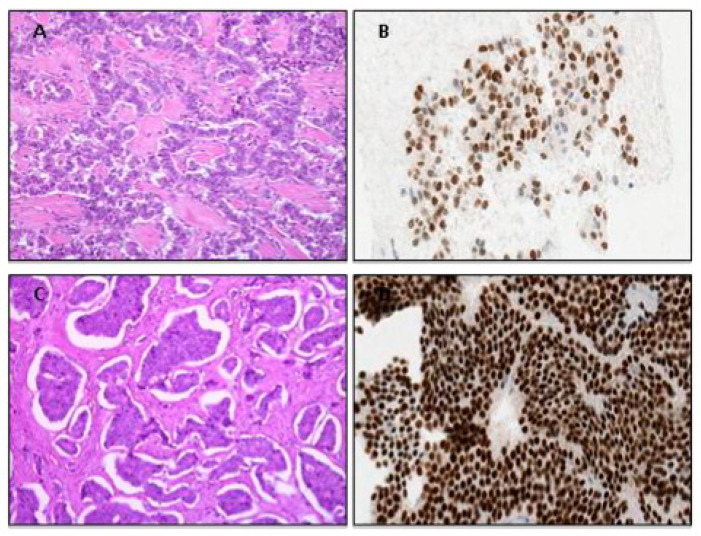
(**A**), Characteristic ribbon-like architectural pattern of pancreatic well-differentiated neuroendocrine tumor with site-specific ISLET-1 immunostaining positivity outline pancreatic landscape (**B**); in contrast, intestinal NENs usually present an organoid morphological pattern (**C**) with immunoreactivity for site-specific marker CDX2 in an intestinal landscape (**D**).

In summary, morphological, immunohistochemical and molecular analyses are equally essential in the assessment of NENs. NENs may exhibit variable growth patterns and cellular characteristics easily identifiable on routine hematoxylin–eosin staining alone [25,26]. For instance, while metastatic NENs with a primary tumor located in the stomach and duodenum may demonstrate a glandular-like pattern, and SI-NENs often exhibit an organoid or nested growth pattern; in contrast, pancreatic and rectal NENs may present with a ribbon-like or trabecular architecture (Figure 3). To identify the actual origin of a UPO-NEN, a wide variety of immunohistochemical markers may be assessed. These include classic markers such as Chromogranin A (CgA) and Synaptophysin (SYP) or INSM1 [27] to confirm the neuroendocrine differentiation [28,29]. CDX2 is a transcription factor, a useful marker of intestinal NENs and, because of its association with GI differentiation, it is also found in gastrin-positive pancreatic NENs and colorectal adenocarcinoma [30,31]. In the setting of WDNENs, Thyroid Transcription Factor1 (TTF1) positivity may suggest a bronchial primary in 43% of the cases. However, it is not specific in poorly differentiated lung neuroendocrine carcinomas (PDNECs), as it is also present in 50% of small-cell tumors at other sites [30,32,33]. Islet-1 (ISL1) can be used as a marker for pancreatic origin [34,35]. Serotonin, associated with CDX2 and SATB2, has utility in identifying EC tumors originating in the ileum or appendix [36,37]. Colorectal NENs may present with positive staining for glucagon-like peptide 1, CDX2 and SATB2 [35,38]. Pheochromocytomas and abdominal paragangliomas stain positive for neuroendocrine markers CgA, SYP, ISL1, INSM1 and, often, GATA3; subsets of cases may display an intricate network of supporting sustentacular cells which are highlighted by an S100 or SOX10 stain [39]. Paranuclear dot-like staining for CgA, CK20 and Neurofilament (NF), and polyomavirus stain, may also help in the identification of neuroendocrine skin lesions such as Merkel cell carcinomas (MCCs) [40,41]. Therefore, to successfully identify UPO-NENs, a combined assessment using clues from clinical history, radiology, morphology and immunohistochemistry is recommended, rather than blind trust in a single marker [12,42]. The interaction between physicians and pathologists is, therefore, fundamental.

#### 3.3.2. Functional Imaging

Somatostatin receptor scintigraphy (SRS) has been extensively used for the initial staging of disease and to evaluate somatostatin receptor (SSTRs) status; furthermore, it has been explored to detect occult primary sites in patients with metastatic gastro-entero-pancreatic (GEP) NENs with a detection rate of 39%. However, 68GaDOTANOC positron emission tomography (PET)/CT has proved to be more accurate and generally represents the functional imaging of choice, being able to also detect very small lesions [43]. According to previous experiences, Ga-68-DOTANOC PET/CT helped in the detection of undiagnosed primary sites in patients with metastatic NENs in a percentage ranging from 45.5% [44] to 59% of the patients [45,46].

A recent meta-analysis [47], including 10 studies of a total of 484 patients with UPO-NENs, demonstrated the high diagnostic sensitivity of 68Ga-DOTA-SSTR for UPO-NENs. 68Ga-DOTA-SSTR PET/CT was highly effective in locating the primary and metastatic sites of UPO-NENs, with a pooled detection rate of 61%.

Fluorodeoxyglucose PET may be employed for the detection of occult primary sites in case of high-grade histology (G3 NEN), whereas F-DOPA and MIBG imaging may be employed in selected cases, especially when paraganglioma/pheochromocytoma are suspected.

#### 3.3.3. Capsule Endoscopy (CE) and Double-Balloon Enteroscopy (DBE)

SI-NENs have always been considered difficult to diagnose in view of their non-specific presentation and poor accessibility of the distal small bowel [48].

Conventional radiology (with or without enteroclysis) is often not accurate enough in the detection of SI-NENs [45], whilst PET/CT with 68Ga-DOTA peptides, despite being the most accurate modality in the detection of well-differentiated NENs, does not allow to obtain a histological diagnosis and might be unable to differentiate between intestinal and mesenteric localization [49].

Capsule endoscopy (CE) and double-balloon enteroscopy (DBE) have significantly improved the diagnosis of SI-NENs, although their use is still limited in routine clinical practice and data on their actual safety and efficacy in the neuroendocrine setting are scant [50]. In a retrospective study [51], in 11 patients with UPO-NEN, CE identified lesions suggestive of small-bowel primary in 8/10 patients in whom it was successful, and all these tumors were histologically confirmed. Conversely, in a recent prospective study by Furnari et al. [52], in 24 patients with a histological diagnosis of metastatic NEN of UPO, the diagnostic yield of CE was compared with the surgical exploration. CE identified a primary SI-NEN in eleven subjects, but the final diagnosis of SI-NEN was confirmed only in five cases after surgical exploration. It is likely that the high number of false-positive results might have been the consequence of confounding factors, including small-bowel contractions, extrinsic compression and lymph stasis.

DBE is more invasive when compared with CE, but allows to determine the precise location as well as the actual number of tumors, and, more importantly, to obtain biopsies for obtaining a pathological diagnosis. In a study involving 12 patients with suspected SI-NEN or with liver NEN metastases, who underwent DBE, a diagnostic yield of DBE for primary tumor of 33% was reported [53]. In five patients with metastatic midgut NENs who underwent DBE, a NEN of the ileum was detected and histologically confirmed in four out of the five patients, whereas conventional radiological imaging did not visualize any of the primary tumors [54]. In a recent prospective study [55], sensitivity and specificity for DBE in detecting SI-NEN were reported to be of 60% and 100%, respectively. According to the available data, DBE should be the technique of choice in the pre-surgical setting given the high specificity, also considering that it allows one to obtain a histological diagnosis. However, further studies are warranted to clarify the actual role of CE and DBE in the diagnostic algorithm of UPO-NENs.

#### 3.3.4. Ultrasound Endoscopy (EUS)

Ultrasound endoscopy (EUS) represents the diagnostic gold standard for pancreatic NENs with an up-to-94% sensitivity [56] and is the technique of choice for the locoregional staging of gastric, duodenal and rectal NENs. Notably, EUS sensitivity in detecting pancreatic NENs is higher than the CT scan or MRI pancreatic lesion detection rate [57]. According to a recent systematic review and meta-analysis, the adjusted incremental benefit of preoperative EUS for the detection of suspected pancreatic NENs after other investigative modalities had failed was 26% and EUS allowed for the identification of pancreatic NENs in 97% of the cases [58].

**Figure 4:**
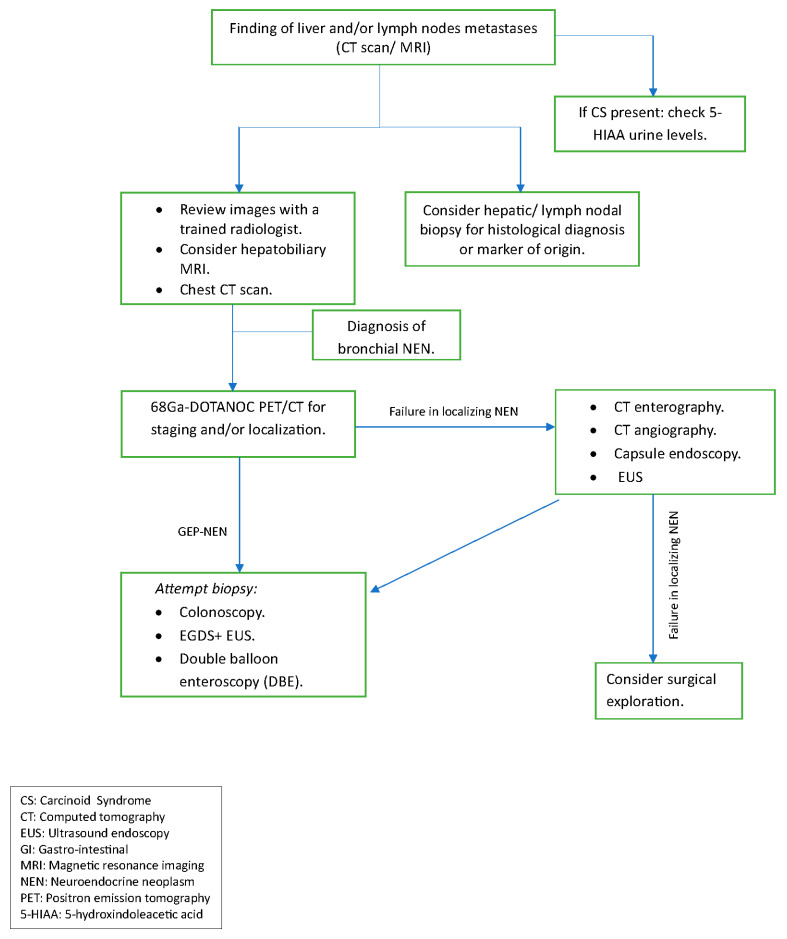
A possible diagnostic algorithm for the detection of neuroendocrine neoplasms of unknown origin.

A possible diagnostic algorithm for the detection of NENs of UPO is proposed in Figure 4.

## 4. Discussion

UPO-NENs constitute 11% to 14% of all GEP-NENs, representing a challenging entity for both diagnosis and treatment. Early-identification of the primary tumor is necessary to define a patient’s management and prognosis [4], taking into account that the resection of the primary tumor even in a metastatic disease is generally correlated with a better survival [6]. This is particularly true for SI-NEN, as the resection of the primary tumor also reduces the risk of complications (i.e., intestinal sub-occlusion/occlusion, abdominal pain due to mesenteric fibrosis), taking into account that this kind of surgery is characterized by low morbidity and mortality. On the other hand, there are fewer clear-cut indications whenever the primary tumor is located in the pancreas and the patient is metastatic, considering the major complications that may follow pancreatic surgery.

The majority of UPO-NENs are found in the small bowel [9,10]. However, localization of midgut tumors might be challenging due to their usually small size and the impaired accessibility of the small bowel to standard endoscopic techniques [48]. The clinical presentation is often non-specific and not particularly useful for diagnosis; however, in the case of functioning tumors, the presence of CS, also diagnosed through elevated levels of 5HIAA, or the presence of symptoms typical of pancreatic/duodenal functioning NENs (see Table 1) might be of help in identifying the site of the primary tumor. In the diagnostic and staging phase, 68GaPET is fundamental. This is particularly true for asymptomatic UPO-NENs and/or whenever pathology is not helpful, also considering its high diagnostic accuracy in the detection of very small lesions [43] and the ability to detect undiagnosed primary sites in patients with metastatic NENs in up to 59% of cases [45,46]. In this scenario, a multimodal approach including conventional radiology and more sophisticated techniques such as CT enterography and CT angiography, nuclear medicine and endoscopy is necessary.

In the specific setting of UPO-NENs, the role of pathology is essential to provide useful information which can orientate clinicians in the search for the primary site. In this context, a wide variety of immunohistochemical markers may be assessed, as previously described. From a practical point of view, if CDX2 is expressed, an intestinal origin is more likely [30], suggesting the need for endoscopic procedures including VCE and DBE in order to find the primary tumor, the latter also allowing to achieve histological confirmation. Whenever Islet-1 (ISL1) is expressed, a pancreatic origin is suggested [34] and, in this case, EUS should be the diagnostic tool of choice, being the gold standard for pancreatic NENs with an up-to-94% sensitivity [56]. Finally, a lymph node or liver metastasis with Thyroid Transcription Factor1 (TTF1) positivity may suggest a bronchial primary in 43% of the cases; however, the role of immunohistochemical markers could be less accurate in poorly differentiated tumors [30,32]. It is, indeed, clear that the proper interaction between clinicians and pathologists is fundamental for the management of these tumors.

As a future perspective, molecular biology and gene-expression profiling represent a promising and growing area in order to obtain a subtype-specific NEN molecular-landscape characterization, that, together with clinical and pathological data, may help to determine tumor origin in UPO-NENs and, even more importantly, might allow one to identify molecular treatment targets.

## 5. Conclusions

In summary, the management of UPO-NENs is challenging, taking into account that therapeutic options are limited. Multidisciplinary management in the diagnostic setting, including a strict cooperation between clinicians and pathologists, plays a key role in this setting, even more than in other NENs. The identification of the primary tumor is warranted, particularly in well-differentiated forms, in order to improve survival and allow access to adequate treatment options. As a matter of fact, once the primary tumor has been removed, generally limiting the disease to the liver, viable curative strategies are available, including liver resection and, in highly selected cases, liver transplantation. Furthermore, some treatment options including targeted therapies and radiopeptide treatment may be limited by registrative boundaries in UPO-NENs. Further studies, possibly prospective and randomized, are needed to improve the management of these tumors, with a specific focus on molecular biology and gene-expression profiling, which might be of great help for both the diagnosis and the treatment of these neoplasms.

## Figures and Tables

**Table 1:** Functioning neuroendocrine neoplasms and their associated clinical syndromes.

**Insulinoma**	**Whipple’s triad** Hypoglycemia (<50 mg/dL) Hypoglycemic symptoms (dizziness, sweating, confusion, increased HF) Symptoms’ resolution with glucose ingestion
**Gastrinoma**	**Zollinger Ellison Syndrome** Peptic ulcer disease Diarrhea Gastro-esophageal reflux disease Weight loss
**VIP-oma**	**Verner Morrison Syndrome** Watery diarrhea Dehydration Hyperkalemia
**Glucagonoma**	Diarrhea Glucose intolerance/diabetes Necrolytic migratory erythema Weight loss and steathorrhoea Anemia
**Somatostatinoma**	Diarrhea Weight loss Diabetes Gallstones

## Data Availability

Not applicable.

## References

[1] Bhosale P., Shah A., Wei W., Varadhachary G., Johnson V., Shah V., Kundra V. (2013). Carcinoid tumours: Predicting the location of the primary neoplasm based on the sites of metastases. Eur. Radiol..

[2] Yao J.C., Hassan M., Phan A., Dagohoy C., Leary C., Abdalla J.E.M.K., Fleming J.B., Vauthey J.-N., Rashid A., Evans D.B. (2008). One hundred years after “carcinoid”: Epidemiology of and prognostic factors for neuroendocrine tumors in 35,825 cases in the United States. J. Clin. Oncol..

[3] Stella G.M., Senetta R., Cassenti A., Ronco M., Cassoni P. (2012). Cancers of unknown primary origin: Current perspectives and future therapeutic strategies. J. Transl. Med..

[4] Stoyianni A., Pentheroudakis G., Pavlidis N. (2011). Neuroendocrine carcinoma of unknown primary: A systematic review of the literature and a comparative study with other neuroendocrine tumors. Cancer Treat. Rev..

[5] Ahmed A., Turner G., King B., Jones L., Culliford D., McCance D., Ardill J., Johnston B.T., Poston G., Rees M. (2009). Midgut neuroendocrine tumours with liver metastases: Results of the UKINETS study. Endocr. Relat. Cancer.

[6] Citterio D., Pusceddu S., Facciorusso A., Coppa J., Milione M., Buzzoni R., Bongini M., Debraud F., Mazzaferro V. (2017). Primary tumour resection may improve survival in functional well-differentiated neuroendocrine tumours metastatic to the liver. Eur. J. Surg. Oncol..

[7] Taal B.G., Visser O. (2004). Epidemiology of Neuroendocrine Tumours. Neuroendocrinology.

[8] Rindi G., Bordi C., Rappel S., La Rosa S., Stolte M., Solcia E. (1996). Gastric carcinoids and neuroendocrine carcinomas: Path-ogenesis, pathology, and behavior. World J. Surg..

[9] Pavel M., O’Toole D., Costa F., Capdevila J., Gross D., Kianmanesh R., Krenning E., Knigge U., Salazar R., Pape U.-F. (2016). ENETS Consensus Guidelines Update for the Management of Distant Metastatic Disease of Intestinal, Pancreatic, Bronchial Neuroendocrine Neoplasms (NEN) and NEN of Unknown Primary Site. Neuroendocrinology.

[10] Dasari A., Shen C., Halperin D., Zhao B., Zhou S., Xu Y., Shih T., Yao J.C. (2017). Trends in the Incidence, Prevalence, and Survival Outcomes in Patients with Neuroendocrine Tumors in the United States. JAMA Oncol..

[11] Abdel-Rahman O. (2021). A Real-World, Population-Based Study for the Incidence and Outcomes of Neuroendocrine Neoplasms of Unknown Primary. Neuroendocrinology.

[12] Berner A.M. (2020). Nostic Approaches to Neuroendocrine Neoplasms of Unknown Primary Site. Neuroendocrinology.

[13] Datta S., Williams N., Suortamo S., Mahmood A., Oliver C., Hedley N., Ray P. (2011). Carcinoid syndrome from small bowel endocrine carcinoma in the absence of hepatic metastasis. Age Ageing.

[14] Halperin D.M., Shen C., Dasari A., Xu Y., Chu Y., Zhou S., Shih Y.-C.T., Yao J.C. (2017). Frequency of carcinoid syndrome at neuroendocrine tumour diagnosis: A population-based study. Lancet Oncol..

[15] Ramage J.K., Ahmed A., Ardill J., Bax N., Breen D.J., Caplin M.E., Corrie P., Davar J., Davies A.H., Lewington V. (2012). Guidelines for the management of gastroenteropancreatic neuroendocrine (including carcinoid) tumours (NETs). Gut.

[16] Alexandraki K., Angelousi A., Boutzios G., Kyriakopoulos G., Rontogianni D., Kaltsas G. (2017). Management of neuroendocrine tumors of unknown primary. Rev. Endocr. Metab. Disord..

[17] Wang S.C., Parekh J.R., Zuraek M.B., Venook A.P., Bergsland E.K., Warren R.S., Nakakura E.K. (2010). Identification of Unknown Primary Tumors in Patients with Neuroendocrine Liver Metastases. Arch. Surg..

[18] Massimino K.P., Han E., Pommier S.J., Pommier R.F. (2012). Laparoscopic surgical exploration is an effective strategy for locating occult primary neuroendocrine tumors. Am. J. Surg..

[19] Spada F., Rossi R.E., Kara E., Laffi A., Massironi S., Rubino M., Grimaldi F., Bhoori S., Fazio N. (2021). Carcinoid Syndrome and Hyperinsulinemic Hypoglycemia Associated with Neuroendocrine Neoplasms: A Critical Review on Clinical and Pharmacological Management. Pharmaceuticals.

[20] Rossi R.E., Elvevi A., Citterio D., Coppa J., Invernizzi P., Mazzaferro V., Massironi S. (2021). Gastrinoma and Zollinger Ellison syndrome: A roadmap for the management between new and old therapies. World J. Gastroenterol..

[21] Wu Y., Xiong G., Zhang H., Wang M., Zhu F., Qin R. (2021). Adrenocorticotropic Hormone-Producing Pancreatic Neuroendocrine Neoplasms: A Systematic Review. Endocr. Pract..

[22] Bergsland E.K., Nakakura E.K. (2014). Neuroendocrine Tumors of Unknown Primary: Is the Primary Site Really Not Known?. JAMA Surg..

[23] Catena L., Bichisao E., Milione M., Valente M., Platania M., Pusceddu S., Ducceschi M., Zilembo N., Formisano B., Bajetta E. (2011). Neuroendocrine tumors of unknown primary site: Gold dust or misdiagnosed neoplasms?. Tumori J..

[24] Pavel M., Baudin E., Couvelard A., Krenning E., Öberg K., Steinmüller T., Anlauf M., Wiedenmann B., Salazar R. (2012). ENETS Consensus Guidelines for the Management of Patients with Liver and Other Distant Metastases from Neuroendocrine Neoplasms of Foregut, Midgut, Hindgut, and Unknown Primary. Neuroendocrinology.

[25] WHO Classification of Tumours Editorial Board (2021). WHO Classification of Tumours. Digestive System Tumours.

[26] WHO Classification of Tumours of Endocrine Organs (2017). World Health Organization Classification of Tumours.

[27] Lilo M.T., Chen Y., LeBlanc R.E. (2018). INSM1 Is More Sensitive and Interpretable than Conventional Immunohistochemical Stains Used to Diagnose Merkel Cell Carcinoma. Am. J. Surg. Pathol..

[28] Oien K.A. (2009). Pathologic Evaluation of Unknown Primary Cancer. Semin. Oncol..

[29] Kriegsmann K., Zgorzelski C., Muley T., Christopoulos P., Thomas M., Winter H., Eichhorn M., Eichhorn F., von Winterfeld M., Herpel E. (2021). Role of Synaptophysin, Chromogranin and CD56 in adenocarcinoma and squamous cell carcinoma of the lung lacking morphological features of neuroendocrine differentiation: A retrospective large-scale study on 1170 tissue samples. BMC Cancer.

[30] Lin X., Saad R.S., Luckasevic T.M., Silverman J.F., Liu Y. (2007). Diagnostic Value of CDX-2 and TTF-1 Expressions in Separating Metastatic Neuroendocrine Neoplasms of Unknown Origin. Appl. Immunohistochem. Mol. Morphol..

[31] Silberg D.G., Swain G.P., Suh E.R., Traber P.G. (2000). Cdx1 and Cdx2 expression during intestinal development. Gastroenterology.

[32] Jagirdar J. (2008). Application of Immunohistochemistry to the Diagnosis of Primary and Metastatic Carcinoma to the Lung. Arch. Pathol. Lab. Med..

[33] Lazzaro D., Price M., Felice M.D., Lauro R.D. (1991). The transcription factor TTF-1 is expressed at the onset of thyroid and lung morphogenesis and in restricted regions of the foetal brain. Development.

[34] Koo J., Mertens R.B., Mirocha J.M., Wang H.L., Dhall D. (2012). Value of Islet 1 and PAX8 in identifying metastatic neuroendocrine tumors of pancreatic origin. Mod. Pathol..

[35] Koo J., Zhou X., Moschiano E., De Peralta-Venturina M., Mertens R.B., Dhall D. (2013). The Immunohistochemical Expression of Islet 1 and PAX8 by Rectal Neuroendocrine Tumors Should Be Taken into Account in the Differential Diagnosis of Metastatic Neuroendocrine Tumors of Unknown Primary Origin. Endocr. Pathol..

[36] Solcia E., Vanoli A. (2014). Histogenesis and Natural History of Gut Neuroendocrine Tumors: Present Status. Endocr. Pathol..

[37] Bellizzi A.M. (2020). SATB2 in neuroendocrine neoplasms: Strong expression is restricted to well-differentiated tumours of lower gastrointestinal tract origin and is most frequent in Merkel cell carcinoma among poorly differentiated carcinomas. Histopathology.

[38] Kim J.Y., Kim K.-S., Kim K.-J., Park I.J., Lee J.L., Myung S.-J., Park Y., Park Y.S., Yu C.S., Kim J.C. (2015). Non-L-cell Immunophenotype and Large Tumor Size in Rectal Neuroendocrine Tumors Are Associated With Aggressive Clinical Behavior and Worse Prognosis. Am. J. Surg. Pathol..

[39] Juhlin C.C. (2021). Challenges in Paragangliomas and Pheochromocytomas: From Histology to Molecular Immunohistochemistry. Endocr. Pathol..

[40] Shuda M., Arora R., Kwun H.J., Feng H., Sarid R., Fernández-Figueras M.-T., Tolstov Y., Gjoerup O., Mansukhani M.M., Swerdlow S.H. (2009). Human Merkel cell polyomavirus infection I. MCV T antigen expression in Merkel cell carcinoma, lymphoid tissues and lymphoid tumors. Int. J. Cancer.

[41] Kuhajda F.P., Olson J.L., Mann R.B. (1986). Merkel cell (small cell) carcinoma of the skin: Immunohistochemical and ultrastructural demonstration of distinctive perinuclear cytokeratin aggregates and a possible association with B cell neoplasms. Histochem. J..

[42] Juhlin C.C., Zedenius J., Höög A. (2022). Metastatic Neuroendocrine Neoplasms of Unknown Primary: Clues from Pathology Workup. Cancers.

[43] Pellegrino F., Granata V., Fusco R., Grassi F., Tafuto S., Perrucci L., Tralli G., Scaglione M. (2023). Diagnostic Management of Gastroenteropancreatic Neuroendocrine Neoplasms: Technique Optimization and Tips and Tricks for Radiologists. Tomography.

[44] Schreiter N.F., Bartels A.-M., Froeling V., Steffen I., Pape U.-F., Beck A., Hamm B., Brenner W., Röttgen R. (2014). Searching for primaries in patients with neuroendocrine tumors (NET) of unknown primary and clinically suspected NET: Evaluation of Ga-68 DOTATOC PET/CT and In-111 DTPA octreotide SPECT/CT. Radiol. Oncol..

[45] Prasad V., Ambrosini V., Hommann M., Hoersch D., Fanti S., Baum R.P. (2010). Detection of unknown primary neuroendocrine tumours (CUP-NET) using 68Ga-DOTA-NOC receptor PET/CT. Eur. J. Nucl. Med. Mol. Imaging.

[46] Pruthi A., Pankaj P., Verma R., Jain A., Belho E.S., Mahajan H. (2016). Ga-68 DOTANOC PET/CT imaging in detection of primary site in patients with metastatic neuroendocrine tumours of unknown origin and its impact on clinical decision making: Experience from a tertiary care centre in India. J. Gastrointest. Oncol..

[47] Ma H., Kan Y., Yang J. (2021). Clinical value of ^68^Ga-DOTA-SSTR PET/CT in the diagnosis and detection of neuroendocrine tumors of unknown primary origin: A systematic review and meta-analysis. Acta Radiol..

[48] Rossi R.E., Conte D., Elli L., Branchi F., Massironi S. (2017). Endoscopic techniques to detect small-bowel neuroendocrine tumors: A literature review. United Eur. Gastroenterol. J..

[49] Sharma P., Arora S., Mukherjee A., Pal S., Sahni P., Garg P., Khadgawat R., Thulkar S., Bal C., Kumar R. (2014). Predictive Value of 68Ga-DOTANOC PET/CT in Patients with Suspicion of Neuroendocrine Tumors: Is Its Routine Use Justified?. Clin. Nucl. Med..

[50] Rossi R.E., Elvevi A., Gallo C., Palermo A., Invernizzi P., Massironi S. (2022). Endoscopic techniques for diagnosis and treatment of gastro-entero-pancreatic neuroendocrine neoplasms: Where we are. World J. Gastroenterol..

[51] Frilling A., Smith G., Clift A.K., Martin J. (2014). Capsule endoscopy to detect primary tumour site in metastatic neuroendocrine tumours. Dig. Liver Dis..

[52] Furnari M., Buda A., Delconte G., Citterio D., Voiosu T., Ballardini G., Cavallaro F., Savarino E., Mazzaferro V., Meroni E. (2017). The Role of Wireless Capsule Endoscopy (WCE) in the Detection of Occult Primary Neuroendocrine Tumors. J. Gastrointestin. Liver Dis..

[53] Bellutti M., Fry L.C., Schmitt J., Seemann M., Klose S., Malfertheiner P., Mönkemüller K. (2009). Detection of Neuroendocrine Tumors of the Small Bowel by Double Balloon Enteroscopy. Dig. Dis. Sci..

[54] Scherübl H., Faiss S., Tschöpe R., Zeitz M. (2005). Double-balloon enteroscopy for the detection of midgut carcinoids. Gastrointest. Endosc..

[55] Rossi R.E., Elli L., Branchi F., Conte D., Massironi S. (2021). Double-Balloon Enteroscopy in Detecting Small-Bowel Neuroendocrine Neoplasms: A Single-Center Prospective Study. Digestion.

[56] Falconi M., Eriksson B., Kaltsas G., Bartsch D.K., Capdevila J., Caplin M., Kos-Kudla B., Kwekkeboom D., Rindi G., Klöppel G. (2016). ENETS Consensus Guidelines Update for the Management of Patients with Functional Pancreatic Neuroendocrine Tumors and Non-Functional Pancreatic Neuroendocrine Tumors. Neuroendocrinology.

[57] Manta R., Nardi E., Pagano N., Ricci C., Sica M., Castellani D., Bertani H., Piccoli M., Mullineris B., Tringali A. (2016). Pre-operative Diagnosis of Pancreatic Neuroendocrine Tumors with Endoscopic Ultrasonography and Computed Tomography in a Large Series. J. Gastrointestin. Liver Dis..

[58] James P.D., Tsolakis A.V., Zhang M., Belletrutti P.J., Mohamed R., Roberts D.J., Heitman S.J. (2015). Incremental benefit of preoperative EUS for the detection of pancreatic neuroendocrine tumors: A meta-analysis. Gastrointest. Endosc..

